# Supplementary material for: Coexistence of distinct skyrmion phases observed in hybrid ferromagnetic/ferrimagnetic multilayers
Source: Nat Commun. 2020 Dec 11;11:6365. doi: 10.1038/s41467-020-20025-2 (PMC7733481; doi:10.1038/s41467-020-20025-2)
Supplement: Supplementary file 1 — Supplementary Information [file 41467_2020_20025_MOESM1_ESM.pdf]

Supplementary Information for

Coexistence of distinct skyrmion phases observed in hybrid ferromagnetic/ferrimagnetic multilayers

Andrada-Oana Mandru[1,*], Oğuz Yıldırım[1], Riccardo Tomasello[2], Paul Heistracher[3], Marcos Penedo[1], Anna Giordano[4], Dieter Suess[3], Giovanni Finocchio[4,†] and Hans Josef Hug[1,5,‡]

[1]*Empa, Swiss Federal Laboratories for Materials Science and Technology, CH-8600 Dübendorf, Switzerland*

[2]*Institute of Applied and Computational Mathematics, FORTH, GR-70013 Heraklion-Crete, Greece*

[3]*Christian Doppler Laboratory for Advanced Magnetic Sensing and Materials, Faculty of Physics, University of Vienna, Boltzmanngasse 5, 1090 Vienna, Austria*

[4]*Department of Mathematical and Computer Sciences, Physical Sciences and Earth Sciences, University of Messina, I-98166 Messina, Italy*

[5]*Department of Physics, University of Basel, CH-4056 Basel, Switzerland*

[*]andrada-oana.mandru@empa.ch
[†]giovanni.finocchio@unime.it
[‡]hans-josef.hug@empa.ch

Supplementary note 1. Magnetometry measurements and material parameters used in the micromagnetic simulations

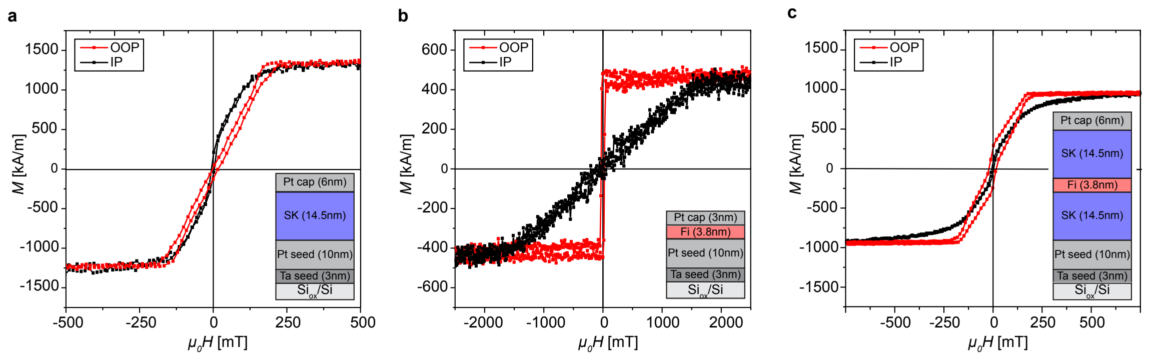

Supplementary Figure 1: **Magnetization loops.** In-plane (IP, black) and out-of-plane (OOP, red) M-H loops acquired at 300 K on **a** the single ferromagnetic skyrmion (SK) layer, **b** the ferrimagnetic (Fi) layer, and **c** the trilayer samples with corresponding layer schematics.

The magnetometry measurements for the single SK layer, the Fi layer, and trilayer samples are presented in Supplementary Fig. 1a-c. Note that all measurements were performed with a magnetic field range of $\pm$ 4 T, well above the saturation field of all samples. However, for clarity purposes, we show the zoomed-in data. The backgrounds coming from the in-plane and out-of-plane holders (together with a Si substrate) were measured independently and then subtracted from all loops. The magnetization values were calculated by considering only the ferromagnetic layers: for the SK layer sample the total thickness $t_{\mathrm{Fe}} + t_{\mathrm{Co}} = 4.5\,\mathrm{nm}$ was considered, and for the trilayer sample the additional $t_{\mathrm{Fe}} + t_{\mathrm{Co}} = 4.5\,\mathrm{nm}$ and the Fi layer thickness of $3.8\,\mathrm{nm}$ were taken into account. From

these measurements, the anisotropy field H_a, saturation magnetization M_s, and the uniaxial perpendicular anisotropy constant K_u for the single SK and Fi layers were extracted and served as part of the input for the micromagnetic simulations.

Layer	SK	Fi
M_s [kA/m]	1371*	488
K_u [kJ/m^3]	1316*	486
A_{ex} [pJ/m]	15	4
D [mJ/m^2]	-2.5	+0.8
$RKKY_{Pt}$ [pJ/m]	4	-

Supplementary Table 1: **Material parameters of the skyrmion (SK) and ferrimagnetic (Fi) layers used in the micromagnetic simulations**. SK = $[Ir(1)/Fe(0.3)/Co(0.6)/Pt(1)]_{\times 5}$ and Fi = $[(TbGd)(0.2)/Co(0.4)]_{\times 6}/(TbGd)(0.2)$; *Note that for the calculation of the magnetization M_s and the anistropy K_u of the SK layer all magnetic moments are attributed to the Fe(0.3)/Co(0.6)-layers.

Supplementary note 2: Skyrmion stray field calculations

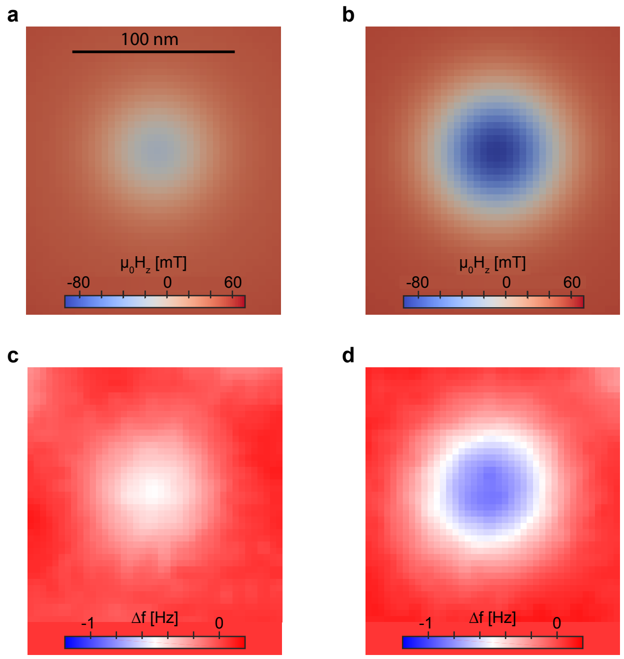

Supplementary Figure 2: **Skyrmion stray field calculations and comparison to magnetic force microscopy data.** z-component of the stray field generated by the incomplete **a** and tubular **b** skyrmion. **c** and **d** Magnetic force microscopy (MFM) frequency shift (Δf) images of an incomplete and a tubular skyrmion, respectively. These particular skyrmions show about the same average contrast of the weak (0.58 Hz) and strong (1.05 Hz) contrast skyrmions visible in Fig. 2**j** of the main manuscript.

Supplementary Fig. 2a and b depict the z-component of the stray field generated by each of the two stable skyrmion states, where the weak skyrmion refers to state 1 in Fig. 3b and the strong skyrmion refers to state 2 in Fig. 3e of the main manuscript. The field is

obtained from micromagnetic simulations and evaluated at a distance of 21 nm above the top-most ferromagnetic layer, in accordance with the 6 nm-thick capping Pt layer and the tip-sample distance of about 15 nm used all MFM measurements shown in this study. As seen from Supplementary Fig. 2a, the H_z field varies between -22.7 mT and 70.9 mT; from Supplementary Fig. 2b we obtain a range between -93.3 mT and 71.4 mT. The 1.76 times larger field difference for the case of the tubular skyrmion agrees well with the 1.81 times higher MFM contrast obtained when comparing the strong skyrmions to the weak ones (from Supplementary Fig. 2c and d).

Supplementary note 3:

Skyrmion diameter and chirality as a function of layer position and D_{Fi}

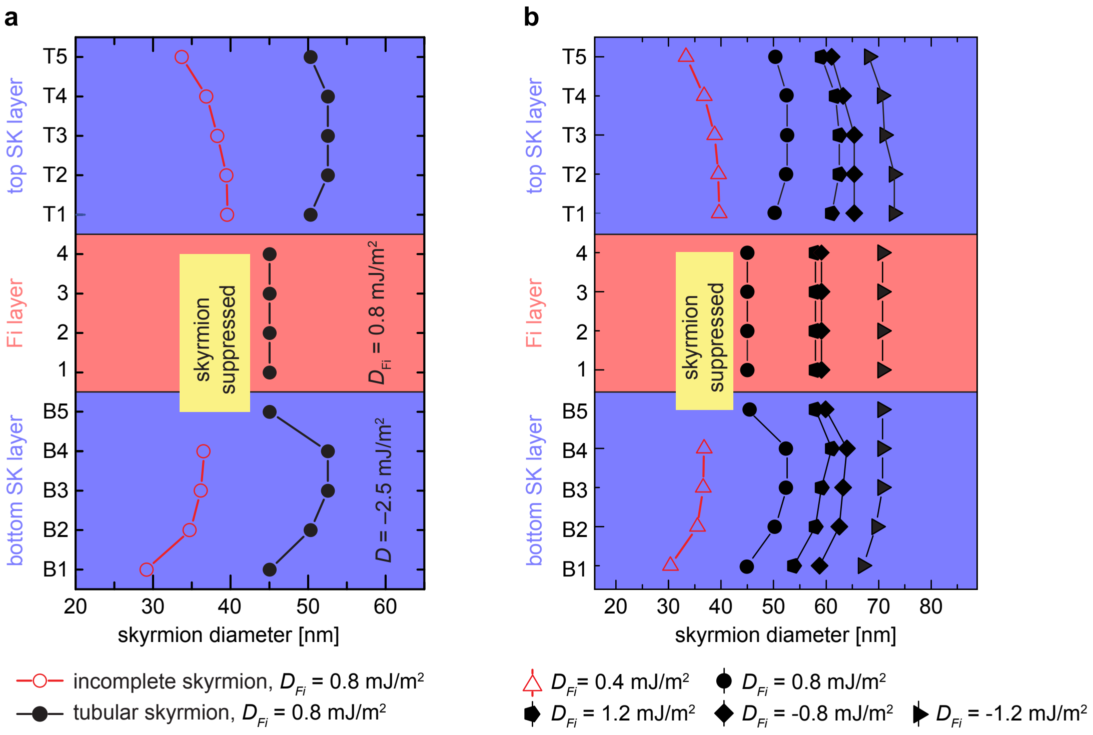

Supplementary Figure 3: **Skyrmion diameter for different D_{Fi} values. a** Skyrmion diameter as a function of layer position for the incomplete and tubular skyrmions; $D = -2.5\,\mathrm{mJ/m^2}$ for the skyrmion (SK) layers and $D_{\mathrm{Fi}} = 0.8\,\mathrm{mJ/m^2}$ for the ferrimagnetic (Fi) interlayer. **b** Skyrmion diameter as a function of layer position and $D_{\mathrm{Fi}} = 0.4\,\mathrm{mJ/m^2}$ (up, open triangles), $0.8\,\mathrm{mJ/m^2}$ (circles), $1.2\,\mathrm{mJ/m^2}$ (pentagons), $-0.8\,\mathrm{mJ/m^2}$ (diamonds), and $-1.2\,\mathrm{mJ/m^2}$ (right triangles).

The skyrmion diameter as a function of layer position and for different values of D_{Fi} are presented in Supplementary Fig. 3. More specifically, Supplementary Fig. 3a shows the

results linked to Fig. 3 of the main text, i.e. $D_{\mathrm{Fi}} = 0.8\,\mathrm{mJ/m^2}$ for an incomplete (open red circles) and a tubular skyrmion (filled black circles). The skyrmion diameters obtained for $D_{\mathrm{Fi}} = 0.4, 0.8, 1.2, -0.8$, and $-1.2\,\mathrm{mJ/m^2}$ are displayed in Supplementary Fig. 3b, where a skyrmion imposed in all the layers has been used as initial state. For $D_{\mathrm{Fi}} = 0.4\,\mathrm{mJ/m^2}$ (open red up-triangles) an incomplete skyrmion is the final state even if a skyrmion spin texture is initially placed in the Fi layer. A negative DMI in the interlayer (the same sign as that of the SK layers) leads to larger skyrmion radii (compare circles to diamonds and pentagons to right-triangles for $D_{\mathrm{Fi}} = \pm 0.8$ and $\pm 1.2\,\mathrm{mJ/m^2}$, respectively). We note the difference in skyrmion diameter between B5/Fi and Fi/T1 interfaces. This is due to the fact that the skyrmions at the lower interface are strongly coupled to the Fi as a result of the large RKKY exchange coupling through the 1 nm of Pt. Since the skyrmions in the top layer are very weakly coupled through the 1 nm of Ir with the Fi, their diameter depends mainly on the SK layer parameters and has therefore a different equilibrium size.

Supplementary Fig. 4 summarizes the spatial distribution of the skyrmion magnetization (equilibrium states) corresponding to the D_{Fi} values reported in Supplementary Fig. 3b. The system is relaxed from an initial configuration where skyrmions with clockwise chirality are placed in all the layers (see Fig. 3d in the main text). The final equilibrium state is the result of a trade-off among negative DMI of the SK layers, different values and signs of the DMI of the Fi layer, magnetostatic interactions, and IEC between

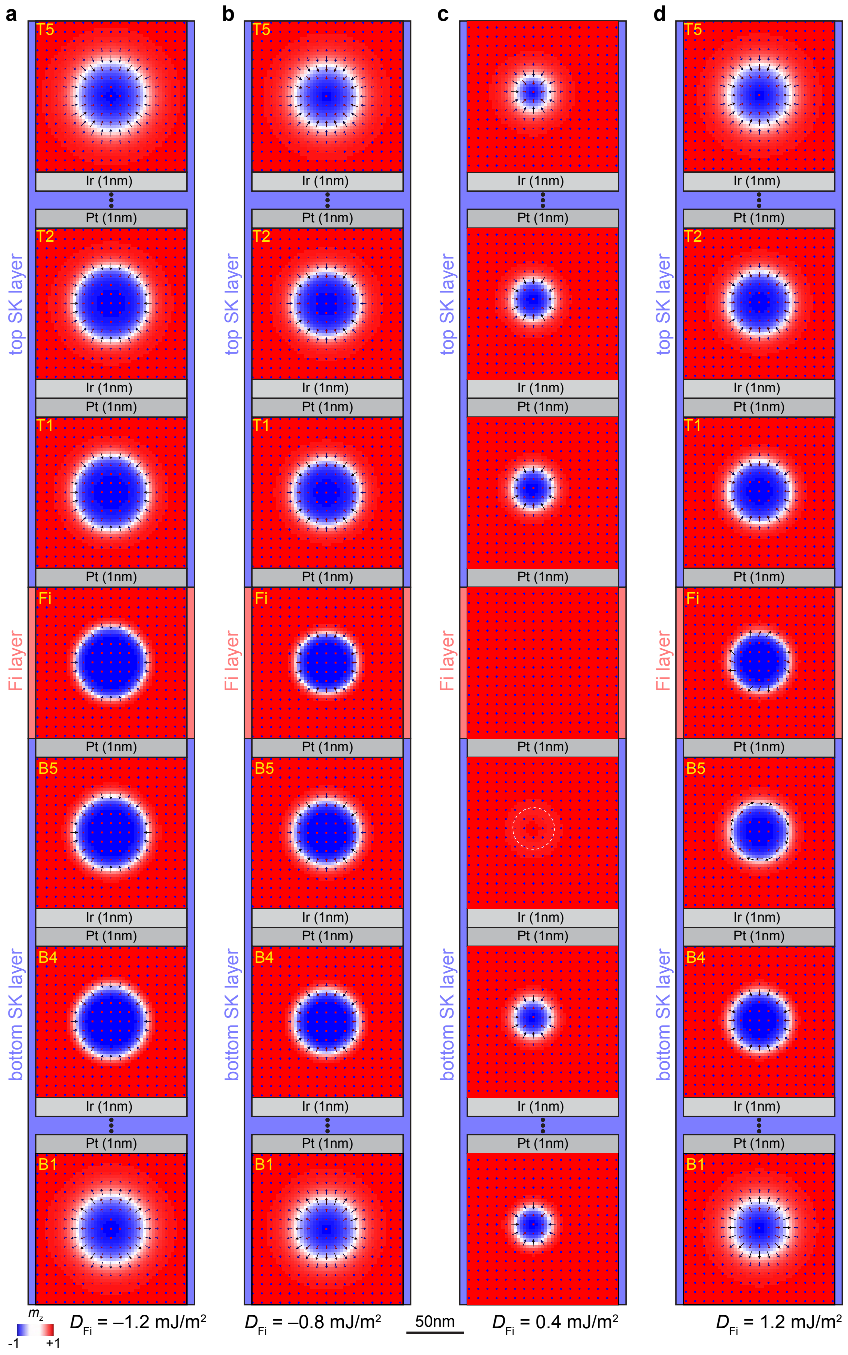

Supplementary Figure 4: **Spatial distribution of the skyrmion magnetization as a function of the sublayer position for different D_{Fi} values for the ferrimagnetic (Fi) interlayer. a** $D_{\mathrm{Fi}} = -1.2\,\mathrm{mJ/m^2}$, **b** $D_{\mathrm{Fi}} = -0.8\,\mathrm{mJ/m^2}$, **c** $D_{\mathrm{Fi}} = 0.4\,\mathrm{mJ/m^2}$, and **d** $D_{\mathrm{Fi}} = 1.2\,\mathrm{mJ/m^2}$.

layer B5 and the first layer of the ferrimagnet.

Supplementary Fig. 4a shows the final state for $D_{\mathrm{Fi}} = -1.2\,\mathrm{mJ/m^2}$. The tubular skyrmion has a large diameter and a clockwise chirality (Néel inward) in layers B2 to B5 and in all sublayers of the top SK layer, as determined by the negative DMI. The counter-clockwise chirality (Néel outward) of the bottom-most layer B1 permits the closure of the magnetic flux driven by the minimization of magnetostatic energy.

In Supplementary Fig. 4b, D_{Fi} is decreased to $-0.8\,\mathrm{mJ/m^2}$, and the effect is only a size reduction of the tubular skyrmion, as also shown in Supplementary Fig. 3b (compare right-triangles to diamonds). When $-0.7\,\mathrm{mJ/m^2} < D_{\mathrm{Fi}} < 0.7\,\mathrm{mJ/m^2}$, the tubular skyrmion is no longer stable and it is replaced by the incomplete skyrmion with a much smaller diameter. Supplementary Fig. 4c depicts an example for $D_{\mathrm{Fi}} = 0.4\,\mathrm{mJ/m^2}$. Note that the chirality now is clockwise in all sublayers of the bottom SK layer. We attribute this to the suppression of the skyrmions in the Fi and in the B5 layers that lowers the in-plane component of the magnetostatic field, thus making the negative DMI the dominant field contribution.

The magnetization of the Fi layer is uniform due to the fact that its DMI is too low to sustain a skyrmion and, consequently, the magnetization of layer B5 is almost

uniform as well (because of the ferromagnetic IEC considered between B5 and the first layer of the ferrimagnet). Indeed, the magnetization of layer B5 has a slight outwards canting at the location highlighted by the white dashed circle in Supplementary Fig. 4c. Such an outwards canting of the magnetization vectors is reminiscent of a skyrmion spin texture with a counter-clockwise chirality (Néel outward) that would occur because of the magnetostatic field from the other layers. In Supplementary Fig. 4d D_{Fi} is increased to $1.2\,\mathrm{mJ/m^2}$, resulting in a tubular skyrmion being stable again. However, because of the opposite signs of the DMI in the Fi and SK layers, a complex dependence of the chirality and type of spin-texture on the vertical position inside the sample occurs, as observed for the tubular skyrmion at $D_{\mathrm{Fi}} = 0.8\,\mathrm{mJ/m^2}$ described in the main text. In layer B1, a skyrmion with a counter-clockwise chirality is obtained because of the minimization of the magnetostatic field. Layers B2-B4 host a skyrmion with clockwise chirality due to the negative DMI of the SK layer. The Bloch skyrmion in the B5-layer compromises between clockwise and counter-clockwise chiralities, which would derive from the negative DMI of the SK layer and from the ferromagnetic IEC with the first layer of the ferrimagnet, respectively. The skyrmion in the Fi layer is intermediate between a Bloch and a Néel with counter-clockwise chiralities. However, the skyrmion here has a chirality closer to the Néel outward due to the larger D_{Fi} with the respect to the case shown in the main text. The skyrmions in the top SK layer maintain a clockwise chirality as favored by the negative

DMI. Our micromagnetic calculations as a function of D_{Fi} suggest that the ferrimagnetic layer should have a sufficiently large DMI in order to allow the coexistence of the two skyrmion phases, otherwise the tubular skyrmion would not be stable.

Supplementary note 4: Results for the coexistence of the two skyrmion phases

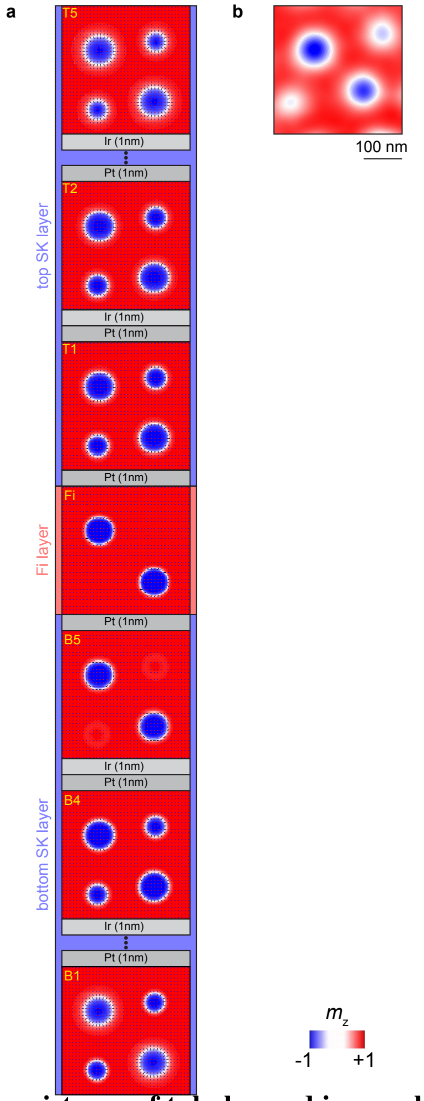

Supplementary Figure 5: **Coexistence of tubular and incomplete skyrmions. a** Spatial distribution of the magnetization showing the coexistence of the two skyrmion states ($D = -2.5\,\mathrm{mJ/m^2}$ for the skyrmion layers and $D_{\mathrm{Fi}} = 0.8\,\mathrm{mJ/m^2}$ for the ferrimagnetic interlayer). **b** Magnetic force microscopy (MFM) sub-image cut from Fig. 1**e** and rotated to resemble the configuration of tubular and incomplete skyrmions of **a**.

Supplementary note 5: Are there skyrmions existing also in only one of the two SK layers?

Our work shows the coexistence of two skyrmions states: i) one showing a strong MFM contrast and consisting of a tubular skyrmion running through all layers (i.e. the bottom SK, the Fi, and the top SK layer); ii) another showing a weaker MFM contrast that exists in the bottom (although, depending on the magnetic field, there is only a partial skyrmion in this bottom layer - see also Supplementary note 6) and top SK layers, and top SK layers but not in the Fi layer. Details regarding the skyrmion structures in the sublayers of the SK layers are shown in Fig. 3 of the main manuscript and discussed in the text. In order to compare the contrast of the two types of skyrmions, only the 23 strongest of the total of 103 weak skyrmions found in Fig. 1f have been analyzed and compared to the total of 23 strong skyrmions found in Fig. 2j. Supplementary Fig. 6a shows the same data as in Fig. 1f but plotted with a color scale reduced to 0.9 Hz from 1.4 Hz to make the weak skyrmions better visible. The red and blue crosses in Supplementary Fig. 6b then shows the center positions of all 23 strong contrast and 103 weak contrast skyrmions visible in Supplementary Fig. 6a. The contrast of these skyrmions is displayed in Supplementary Fig. 6c. The black horizontal line and the grey box show the contrast of the skyrmions found for a SK layer grown on top of the Fi layer (see Fig. 2b and the corresponding MFM data in Fig. 2g). The skyrmion contrast in this layer is only 0.24 ± 0.04 Hz. Note

that this contrast would become even smaller if the skyrmions existing only in the bottom SK layer (which is farther away from the MFM tip) were considered. In Supplementary Fig. 6c only about 8 of the 103 weak-contrast skyrmions have a contrast compatible with skyrmions existing solely in the top SK layer. Therefore, we conclude that most of the weak skyrmions visible in Figs. 1b-h consist of tubular skyrmion existing in the bottom SK and top SK layer but not in the Fi layer.

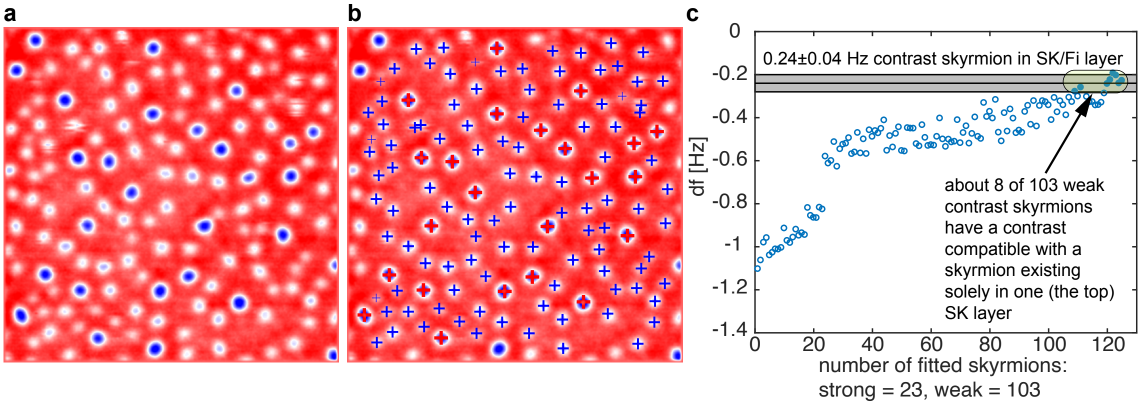

Supplementary Figure 6: **Further analysis on the data displayed in Fig. 1f of the main text.** **a** Skyrmions at 147mT from Fig. 1**f** in the main manuscript, but displayed with a contrast scale of 0.9 Hz to make the weak skyrmions better visible. **b** Same image as in **a**, but with added crosses: red crosses indicate the positions of the 23 skyrmions showing a strong contrast that have been evaluated in Figs. 2**j** and **o**; blue crosses indicate the 103 skyrmions showing a weak (thicker) or very weak (fainter). **c** The results for all fitted skyrmions from **b**. As in Fig. 2**j**, the contrast of the 23 strong skyrmions is noticeably different from that of the 103 weak skyrmions. Only 8 of the total 103 weak skyrmions have a contrast that is compatible with the skyrmions found in the single skyrmion layer sample grown on the top of the ferrimagnetic layer (see schematics in Fig. 2**b** and magnetic force microscopy (MFM) image in Fig. 2**g** of the main manuscript).

Supplementary note 6: Conversion from tubular skyrmions to incomplete skyrmions for higher magnetic fields

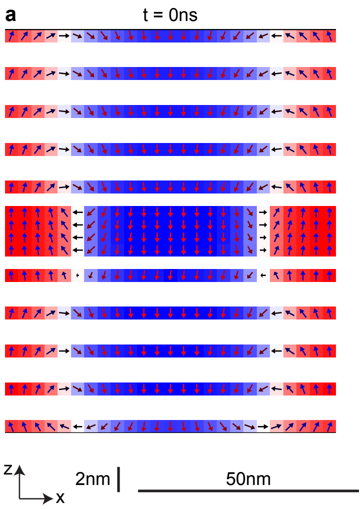

Supplementary Figure 7: **Cross-sectional view of the tubular to incomplete skyrmion conversion in a field of 150 mT and for different time instants. a** 0 ns, **b** 0.8 ns, **c** 50 ns.

Starting with an initial state of the tubular skyrmion shown in Fig. 3f of the main text (Supplementary Fig. 7a here), we carried out additional micromagnetic simulations in which we applied a larger external field. Supplementary Fig. 7 shows the cross-sectional view of the conversion from the tubular skyrmion to the incomplete skyrmion for a field of 150 mT at different time instants. The conversion process occurs via a gradual reduction of the skyrmion diameter, followed by the annihilation of the skyrmion in the Fi layer (Supplementary Fig. 7b), but we also observe the subsequent annihilation of the skyrmion in the first (B1) and last (B5) layers of the bottom SK layer (Supplementary Fig. 7c). For

fields lower than this value, the conversion does not occur, but the role of the field is only to reduce the tubular skyrmion diameter. We can conclude that, as expected, the external field drives the tubular to incomplete skyrmion conversion accompanied by a reduction of the diameter.
